# Influence of fiber degradation and concentration of fermentable sugars on simultaneous saccharification and fermentation of high-solids spruce slurry to ethanol

**DOI:** 10.1186/1754-6834-6-145

**Published:** 2013-10-08

**Authors:** Kerstin Hoyer, Mats Galbe, Guido Zacchi

**Affiliations:** 1 Department of Chemical Engineering, Lund University, P.O. Box 124, Lund SE-221 00, Sweden

**Keywords:** SSF, Simultaneous saccharification and fermentation, Fuel ethanol, High dry matter, High solids, Prehydrolysis, Pre-hydrolysis

## Abstract

**Background:**

Saccharification and fermentation of pretreated lignocellulosic materials, such as spruce, should be performed at high solids contents in order to reduce the cost of the produced bioethanol. However, this has been shown to result in reduced ethanol yields or a complete lack of ethanol production. Previous studies have shown inconsistent results when prehydrolysis is performed at a higher temperature prior to the simultaneous saccharification and fermentation (SSF) of steam-pretreated lignocellulosic materials. In some cases, a significant increase in overall ethanol yield was reported, while in others, a slight decrease in ethanol yield was observed. In order to investigate the influence of prehydrolysis on high-solids SSF of steam-pretreated spruce slurry, in the present study, the presence of fibers and inhibitors, degree of fiber degradation and initial fermentable sugar concentration has been studied.

**Results:**

SSF of whole steam-pretreated spruce slurry at a solids content of 13.7% water-insoluble solids (WIS) resulted in a very low overall ethanol yield, mostly due to poor fermentation. The yeast was, however, able to ferment the washed slurry and the liquid fraction of the pretreated slurry. Performing prehydrolysis at 48°C for 22 hours prior to SSF of the whole pretreated slurry increased the overall ethanol yield from 3.9 to 62.1%. The initial concentration of fermentable sugars in SSF could not explain the increase in ethanol yield in SSF with prehydrolysis. Although the viscosity of the material did not appear to decrease significantly during prehydrolysis, the degradation of the fibers prior to the addition of the yeast had a positive effect on ethanol yield when using whole steam-pretreated spruce slurry.

**Conclusions:**

The results of the present study suggest that the increase in ethanol yield from SSF when performing prehydrolysis is a result of fiber degradation rather than a decrease in viscosity. The increased concentration of fermentable sugars at the beginning of the fermentation phase in SSF following prehydrolysis did not affect the overall ethanol yield in the present study.

## Background

Ethanol can be produced from spruce by enzymatic hydrolysis using cellulolytic enzymes followed by fermentation with baker’s yeast (*Saccharomyces cerevisiae*). Performing these two steps simultaneously in so-called simultaneous saccharification and fermentation (SSF) has shown advantages such as reduced end product inhibition of the cellulolytic enzymes, increased overall ethanol yield, and reduced reactor volume [1-3].

Historically, in most research studies rather low solids loadings, usually between 2-10 wt%, have been employed. In batch SSF of steam-pretreated spruce with 10% water-insoluble solids (WIS) it is possible to achieve almost complete conversion of the fermentable sugars in the substrate to ethanol, resulting in a final ethanol concentration after SSF of about 40 g/L [4-6]. In the production of ethanol from lignocellulosic material such as spruce, it is essential to reach a final ethanol concentration after the fermentation step of at least 4-5 wt% in order to reduce the energy demand, and thus the cost, of product recovery by distillation [7]. The only way to increase the final ethanol concentration is to increase the concentration of fermentable sugars in the process. This can be done by increasing the substrate loading. Recent studies on high solids hydrolysis and fermentation of lignocellulosic material to ethanol often focus on solids concentrations above 10%. However, the use of substrates with high solids contents suffers from various problems, including poor stirring of the material, and high inhibitor concentrations causing inhibition of the yeast, and sometimes also the enzymes [5,8-14]. We have previously found that batch SSF of steam-pretreated spruce with 13.7% WIS resulted in an overall ethanol yield of only 5-6%, even when using reactors modified to handle solid or semi-solid material, thus assuring adequate mixing of the material [8]. We also showed that this process could be improved by performing prehydrolysis at a higher temperature prior to SSF. Prehydrolysis at 48°C for 22 hours increased the final ethanol concentration from 3 to 48 g/L using whole steam-pretreated spruce slurry with 13.7% WIS [8].

The aim of prehydrolysis prior to SSF is to partially hydrolyze the cellulose prior to the addition of the yeast. The optimal temperature range for the cellulolytic enzymes used in this study (Cellic CTec2) is 45-50°C [15], which is higher than the optimal temperature for *S. cerevisiae*. Separate prehydrolysis enables enzymatic hydrolysis to take place at a higher temperature than fermentation. Also, prehydrolysis has been reported to decrease the viscosity of the material, thus facilitating mixing [9,16-18]. In batch SSF, the concentration of fermentable sugars is kept very low during most of the process, which could result in yeast starvation. This could be circumvented by performing prehydrolysis prior to SSF, in order to increase the concentration of fermentable sugars. However, high sugar concentrations can lead to high osmotic pressure in the fermentation medium, which is known to stress the yeast. The incubation of yeast in a medium containing high concentrations of different fermentable sugars has been shown to increase the fermentation ability of the yeast [19]. Thus, high initial concentrations of fermentable sugar as a result of prehydrolysis prior to SSF may influence the fermentation step in the process in various ways.

Performing prehydrolysis at 45-48°C prior to SSF of steam-pretreated spruce, and other lignocellulosic materials, at low solids loadings has not shown any positive effect on ethanol yield, and in some cases even resulted in lower ethanol yields than SSF without prehydrolysis [5,20,21]. Manzanares *et al.*[12] reported no increase in ethanol yield from dilute-acid-pretreated olive tree prunings, even at solids concentrations up to 23% dry matter (DM), whereas they observed an increase in overall ethanol yield from 11 to 50% in SSF of hot-water-pretreated olive prunings with 23% DM when SSF was preceded by prehydrolysis. Prehydrolysis of hot-water-pretreated olive prunings at lower solids concentrations did not increase the ethanol yield [12]. It thus appears that including prehydrolysis prior to SSF does not always improve the overall ethanol yield of SSF and the presence of fiber in SSF might have an effect on the ethanol yield. Previous studies also suggest that not only the initial WIS content influences the overall ethanol yield in SSF. Also, the pretreatment method used prior to SSF has an influence on the overall ethanol yield in SSF [12]. This is partly due to different degrees of degradation of the biomass during pretreatment. Lignocellulosic biomass is further degraded and chemically altered during a prehydrolysis step, which might influence the ethanol yield in the following enzymatic hydrolysis and fermentation.

Although the higher temperature in the prehydrolysis step favors enzymatic hydrolysis, the long retention time in hydrolysis and SSF is believed to result in deactivation of the cellulolytic enzymes [20,21].

Adding a prehydrolysis step prior to SSF thus affects the process in many different ways but this has to our knowledge not been studied intensively, especially not for spruce. We have therefore performed a study of the influence of the degree of fiber degradation, initial monomeric sugar concentration and influence of fibers and inhibitors on SSF of steam-pretreated spruce with a solids loading of 13.7% WIS.

## Results and discussion

The various SSF experiments are presented in Table 1. Unless otherwise stated, the ethanol yields given are the yield from the SSF step, or the yield from prehydrolysis and SSF (SSF 4 and 9), expressed as the percentage of the theoretical, based on the content of glucose and mannose in the liquid and solid material added to the fermentor.

**Table 1:** Summary of the experiments carried out

**SSF run (120 h, 32°C)**	**Substrate**	**Prehydrolysis (48°C)**	**Addition of enzyme**	**Initial sugar concentration**^**a**^**(g/L)**
				
1^b^	Whole slurry	No	Yes	38
2	Washed slurry	No	Yes	36
3	Liquid fraction	No	Yes	39
4^b^	Whole slurry	22 hours	Yes	83^c^
5	Washed slurry	No	Yes	116
6	Washed slurry	No	No	116
7	Washed slurry	No	Yes	76
8	Whole slurry	No	No	61
9^b^	Whole slurry	4 hours	Yes	69^c^
10	Whole slurry	No	No	119

^a^Fermentable sugars (glucose and mannose) in monomeric form.

^b^Adapted from Hoyer *et al.*[8].

^c^Concentration of fermentable sugars (glucose and mannose) at the time of the addition of the yeast.

### Effects of fibers, inhibitors, and prehydrolysis on batch SSF

**Figure 1:**
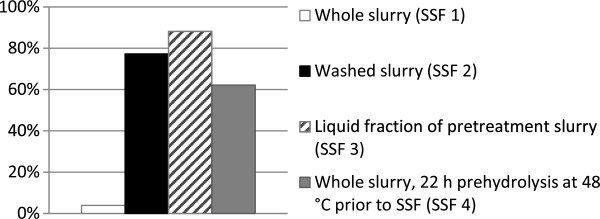
**Overall ethanol yield from SSF and combined prehydrolysis and SSF of spruce slurry with 13.7% WIS.** SSF 2 was compensated for the loss of fermentable sugars in the washing step.

Batch SSF was performed with 13.7% WIS using the whole pretreated spruce slurry (SSF 1), washed pretreated spruce slurry (SSF 2), and the liquid fraction of the pretreated spruce slurry (SSF 3) at a concentration corresponding to that of SSF using whole slurry with 13.7% WIS. The results are presented in Figure 1. SSF of the whole steam-pretreated slurry (SSF 1) resulted in a very low overall ethanol yield (3.9%). Washing the slurry prior to SSF, to remove the inhibitors present in the raw material and those formed in the pretreatment step (SSF 2), increased the overall ethanol yield to 77.2%, suggesting that high viscosity, and thus stirring difficulties, was not the limiting factor in SSF 1. It has previously been shown that inadequate stirring at high solids concentrations can sometimes explain a lower ethanol yield in SSF [5]. The results in this study are in accordance with those of Lu *et al.*[11], who reported almost complete fermentation in SSF of steam-pretreated corn stover at total solids concentrations up to 30% using washed fiber, while the whole pretreated and hydrolyzed slurry could not be fermented at all at the same solids concentration. The glucose concentration in SSF 1 increased throughout the entire SSF process, reaching a final concentration of 68.2 g/L, indicating problems in fermentation. This is in accordance with the results presented by Lu *et al.*[11], who found that no ethanol was produced using whole slurry with DM contents above 20% TS, while the ethanol yield in experiments using washed slurry was not affected by the DM content. They did, however, not see any significant decrease in glucose yield in enzymatic hydrolysis with increasing dry matter content up to 30% TS, using washed and whole steam-pretreated corn stover. It is well known that compounds present in the pretreatment hydrolysate inhibit *S. cerevisiae* in the production of ethanol from lignocellulosic biomass [22-25].

Despite the indications of inhibited fermentation in SSF 1 in the present study, the liquid fraction of the pretreated slurry, was fermented well at a concentration corresponding to 13.7% WIS (SSF 3), resulting in an overall ethanol yield of 88.1%. This indicates that yeast inhibitors such as furfural, hydroxymethylfurfural (HMF) and the organic acids acetic acid and lactic acid were present at low concentrations, and did not lead to any significant inhibition of the yeast with respect to final ethanol yield under the conditions used in the present study (for exact concentrations in the pretreated hydrolysate, see Table 2). This is in accordance with previous findings where acetic acid at concentrations up to around 6 g/L increased the ethanol yield after fermentation of dilute acid pretreated spruce hydrolysate and only inhibited the yeast at higher concentrations [26]. Also furfural and HMF at concentrations higher than the ones in the present study have been shown to cause a lag phase in fermentation, but not to affect the final ethanol yield [26]. It is, however, important to keep in mind that the inhibition of the yeast is depending on the sum of inhibiting substances, but the results in the present study suggest that inhibition of the yeast can be ruled out as the sole explanation of the low ethanol yield in batch SSF with the whole pretreated slurry (SSF 1). As we have shown previously, adding a prehydrolysis step prior to SSF of the whole steam-pretreated spruce slurry with 13.7% WIS at 48°C for 22 hours (SSF 4) resulted in an increase in final ethanol concentration from 3.0 to 47.8 g/L [8]. It is well known that yeast suffers stress as a result of high osmotic pressure or high concentrations of organic acids, and that these factors can act synergistically [27]. The results discussed above (SSF 1-4) show that a combination of high WIS concentration and inhibitors is responsible for the difference in ethanol yield in high-solids batch SSF, while the yeast is able to deal with each one separately.

**Table 2:** Composition of the liquid fraction of the pretreated material (the fraction of sugars present in monomeric form is presented in parentheses as % of the total)

**Component**	**Concentration (g/L)**
Glucose	34.8 (83.8)
Mannose	28.6 (87.8)
Xylose	13.2 (91.5)
Galactose	6.6 (84.6)
Arabinose	0
HMF	1.8
Furfural	2.1
Lactic acid	0
Acetic acid	5.0

The reduction in viscosity resulting from prehydrolysis is often cited as the main reason for adding such a step to SSF [9,11,17,18]. The washed steam-pretreated spruce slurry in the present study was almost completely fermented (SSF 2), despite the fact that this slurry appeared to have the same initial viscosity as in batch SSF using the whole pretreated slurry (SSF 1), which did not yield any significant amount of ethanol. This indicates that prehydrolysis may have other significant effects on the substrate, making it more easily fermented. Jørgensen *et al.*[9] hydrolyzed steam-pretreated wheat straw at total solids concentrations between 20 and 40% and reported that the material changed from a solid to a liquid after about 4 hours. In the present study, no such rapid liquefaction was observed, and the material was only slightly more liquefied after 22 hours of prehydrolysis. It has previously been reported by Palmqvist and Lidén [14] that different lignocellulosic materials behave differently during enzymatic hydrolysis. They observed that steam-pretreated *Arundo donax* (giant cane) quickly lost most of its fiber structure during enzymatic hydrolysis (up to 20% WIS), while the fiber network in spruce was retained for a longer period of time during hydrolysis under the same conditions. It is therefore important to be aware of the fact that different lignocellulosic materials may respond in different ways to prehydrolysis.

Apart from reducing the viscosity, prehydrolysis also results in an increase in the concentration of soluble monomeric sugars, mostly glucose, and the degradation of the fiber structure. In the present study, the concentrations of inhibitors such as furfural, HMF and organic acids (acetic and lactic acid) did not change during prehydrolysis when using steam-pretreated spruce. Since the rapid liquefaction of the material during prehydrolysis reported for agricultural lignocellulosic materials [9,14] was not observed in the present study, and the concentrations of inhibitors were unaffected by prehydrolysis, we investigated whether the degree of degradation of the pretreated material or the initial concentration of fermentable sugars in the SSF step could explain the significant increase in overall ethanol yield in SSF of whole steam-pretreated spruce slurry when adding a prehydrolysis step.

### SSF with varying degree of fiber degradation

**Figure 2:**
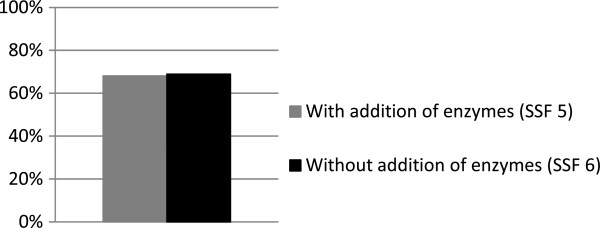
**Overall ethanol yield from SSF of washed steam-pretreated spruce slurry with 13.7% WIS and an initial concentration of glucose of 116 g/L.** In SSF 6, the ethanol yield was compensated for the fact that monomeric sugars were not released from the cellulose structure, since no cellulolytic enzymes were present.

To study the influence of the degree of degradation of the pretreated spruce fibers, SSF was performed on washed pretreated slurry with 13.7% WIS with and without the addition of cellulolytic enzymes (SSF 5 and 6). The overall ethanol yields were 68.1% (with enzymes) and 68.9% (without enzymes), assuming a fermentation yield of 90% to compensate for the fact that new monomeric sugars were not released in SSF 6, where no cellulolytic enzymes were present (Figure 2). In SSF 6, the fibers remained undegraded throughout the entire fermentation period of 120 hours, while in SSF 5 the fibrous material was degraded by enzymatic hydrolysis. The results of SSF 5 and 6 show that the yeast was able to ferment the sugars to ethanol in the presence of undegraded fibers at 13.7% WIS when washed steam-pretreated spruce slurry was used.

**Figure 3:**
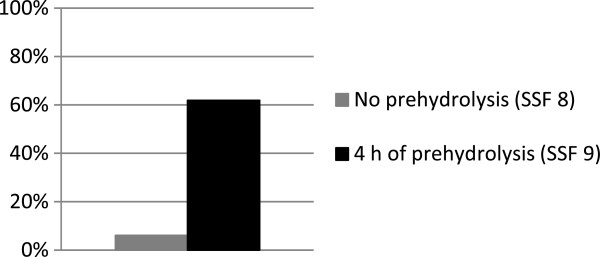
Overall ethanol yield from SSF of whole steam-pretreated spruce slurry with 13.7% WIS and an initial concentration of fermentable sugars of 76 (SSF 8) and 69 g/L (SSF 9).

To study the influence of the degree of degradation when using whole pretreated slurry, SSF was performed with the same initial monomeric sugar concentration (SSF 8) as found after 4 hours of prehydrolysis (SSF 9). The overall ethanol yield was only 6.1%, while SSF following 4 hours of prehydrolysis (SSF 9) resulted in an overall ethanol yield of 57.0% (Figure 3). The only difference between these two experiments was that the material in SSF 9 was partially hydrolyzed at the time of yeast addition, while in SSF 8 the fiber structure was intact at the beginning of SSF. This indicates that the degree of fiber degradation has a major influence on the production of ethanol by SSF when using the whole pretreated spruce slurry at 13.7% WIS.

We used the term “degree of degradation” in this study since the positive effect of prehydrolysis prior to SSF of steam-pretreated spruce slurry appears to originate from something other than a reduction in viscosity during prehydrolysis. It is, however, important to bear in mind that in this study the term degree of degradation should be interpreted in a broad sense, and further studies are required to determine the reason for the difference in overall ethanol yield between degraded and undegraded fiber slurry when using whole steam-pretreated spruce slurry. It is possible that the higher ethanol yield is due to the liberation of compounds from the fiber structure during hydrolysis, and not only from the degradation of the fiber structure.

### SSF with varying initial concentrations of fermentable sugars

**Figure 4:**
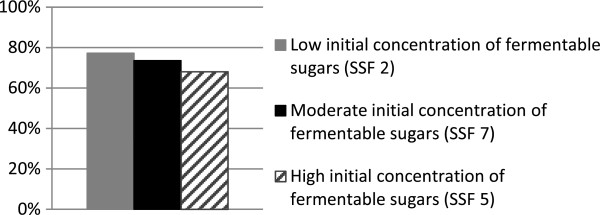
Overall ethanol yield from SSF of washed steam-pretreated spruce slurry with 13.7% WIS, containing different initial amounts of fermentable sugars.

To study the influence of the initial concentration of fermentable sugars (glucose and mannose), SSF of washed pretreated spruce slurry was performed with the addition of different amounts of glucose (SSF 2, 5 and 7). The initial concentrations of fermentable sugars were chosen to include a range from the concentration present in a standard batch SSF with whole pretreated spruce slurry, to concentrations well above those after 4 and 22 hours of prehydrolysis, denoted Low (36-38 g/L), Moderate (61-76 g/L) and High (116-119 g/L). The initial concentrations of fermentable sugars in the different SSF experiments are presented in Table 1. SSF of washed pretreated spruce slurry resulted in overall ethanol yields between 68.1 and 77.2%; the lowest ethanol yield being obtained in SSF of the substrate with highest initial concentration of fermentable sugars (see Figure 4). Hyperosmotic stress due to high sugar concentrations results in the accumulation of intracellular glycerol in yeast cells [19,28], which could explain the slight decrease in ethanol yield at the highest glucose concentrations. The differences in the ethanol yields from SSF of washed steam-pretreated spruce slurry with different initial concentrations of fermentable sugars are not sufficient to explain the difference in ethanol yields observed in batch SSF of whole steam-pretreated spruce slurry with and without prehydrolysis (Figure 3).

**Figure 5:**
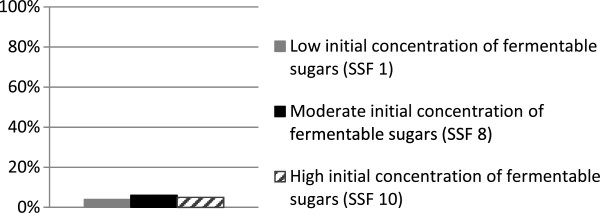
Overall ethanol yield from SSF of whole steam-pretreated spruce slurry with 13.7% WIS, containing different initial amounts of fermentable sugars.

When performing SSF on the whole pretreated spruce slurry spiked with different amounts of glucose (SSF 1, 8 and 10), the overall ethanol yields were very low, between 3.9 and 6.1%, and the initial concentration of fermentable sugars did not affect the overall ethanol yield significantly (Figure 5).

## Conclusions

We have shown in the present study that it is possible to achieve high overall ethanol yields (77.2%) in SSF of washed steam-pretreated spruce slurry at a solids concentration of 13.7% WIS, as well as in SSF of the liquid fraction at a concentration corresponding to 13.7% WIS (88.1%). However, batch SSF of the whole steam-pretreated slurry at the same solids concentration resulted in a final ethanol concentration of only 3.0 g/L. This was, however, increased to 47.8 g/L when prehydrolysis was carried out at 48°C for 22 hours prior to SSF.

Batch SSF experiments using both washed and whole steam-pretreated spruce slurry with different initial sugar concentrations indicate that the initial concentration of fermentable sugars at the time of yeast addition cannot explain the difference in ethanol yields with and without prehydrolysis. Furthermore, the viscosity of the spruce slurry did not appear to decrease significantly during the first few hours of prehydrolysis, and the results of this study therefore suggest that the degradation of fiber during prehydrolysis is the reason for the increase in ethanol yield from the whole steam-pretreated spruce slurry when employing prehydrolysis. When using washed slurry, the yeast was able to ferment the sugars to ethanol, regardless of whether the fiber fraction had been degraded.

In the present study, the term “fiber degradation” was used in a broad sense, and more research is needed to investigate the exact impact of prehydrolysis on the overall process. From the factors investigated in the present study, the significant increase in overall ethanol yield in SSF of whole steam-pretreated slurry with prehydrolysis could be coupled with the degradation of the fiber during prehydrolysis. Whether it, however, actually is structural degradation of the fiber or other, indirect, effects occurring simultaneously during prehydrolysis, remains to be seen. These indirect effects during fiber degradation could be the release of compounds from the material affecting inhibition or steric hindrance during enzymatic hydrolysis or fermentation.

## Methods

### Raw material and steam pretreatment

Unbarked spruce chips were impregnated with 5% SO_2_ and pretreated by continuous steam explosion at 205°C for 6-7 min at SEKAB E-Technology (Örnsköldsvik, Sweden). The pretreated slurry was stored at our laboratory at room temperature in airtight containers. Once opened, the containers were stored at 4°C before analysis and SSF.

The steam-pretreated slurry used in this study had a DM content of 16.4% WIS (24.3% total solids (TS)). The washed pretreated fiber consisted of 49.0% glucan and 48.2% lignin. The composition of the liquid fraction of the pretreated material is presented in Table 2.

### Cell cultivation

#### Inoculum

The inoculum culture was prepared on an agar plate from pure baker’s yeast (*Saccharomyces cerevisiae*) produced by Jästbolaget, Rotebro, Sweden. The cells were added to a 300 mL Erlenmeyer flask together with 70 mL of an aqueous solution containing 23.8 g/L glucose, 10.8 g/L (NH_4_)_2_SO_4_, 5.0 g/L KH_2_PO_4_, and 1.1 g/L MgSO_4_∙7H_2_O. This solution also contained 14.4 g/L trace metal solution and 1.4 g/L vitamin solution, prepared according to Taherzadeh *et al.*[29]. The pH was adjusted to 5 with 0.25 M NaOH, and the Erlenmeyer flask was sealed with a cotton plug and incubated at 30°C for 23 h on a rotary shaker.

### Aerobic cultivation

Aerobic cell cultivation was performed in two steps. Cells were first cultivated in batch mode on a glucose solution, after which the mode was changed to fed-batch with a feed containing hydrolysate liquid from the pretreatment step. Adapting the yeast cells to pretreatment hydrolysate has previously been shown to improve resistance to the inhibitors in the fermentation medium, and to give higher ethanol yields, especially at higher DM contents [30]. Both steps were performed in 2 L fermentors (Infors AG, Bottmingen, Switzerland) at 30°C. The pH was maintained at 5 by the addition of 2.5 M NaOH throughout cell cultivation.

The working volume of batch cultivation was 500 mL. Cultivation was started by adding 60 mL inoculum to a medium containing 20.0 g/L glucose, 22.5 g/L (NH_4_)_2_SO_4_, 10.5 g/L KH_2_PO_4_, 2.2 g/L MgSO_4_∙7H_2_O, 60 g/L trace metal solution, and 6.0 g/L vitamin solution. Batch cultivation was performed at a stirrer speed of 700 rpm. The fermentor was aerated by the addition of air. The air flow was controlled to give a concentration of dissolved oxygen above 5% at all times.

Batch cultivation was changed to fed-batch cultivation when the concentration of dissolved oxygen increased rapidly, showing that all the sugar and the ethanol produced during batch cultivation had been depleted. This occurred after 11-15 hours of batch fermentation in the present study. Fed-batch cultivation was performed with hydrolysate from the pretreatment step. Glucose was added to the hydrolysate to obtain a concentration of fermentable sugars (glucose and mannose) of 70 g/L. A total volume of 1 L feed containing hydrolysate supplemented with salt solution, resulting in feed concentrations of 11.3 g/L (NH_4_)_2_SO_4_, 5.3 g/L KH_2_PO_4_, and 1.1 g/L MgSO_4_∙7H_2_O, was added over a period of 23-24 hours. The final concentration of hydrolysate in the fermentor was equivalent to that which would have been obtained if the slurry from pretreatment had been diluted to 7.5% WIS. Fed-batch cultivation was performed in the aerated fermentor at a stirrer speed of 900-1000 rpm.

### Cell harvest

The cultivation medium was centrifuged in 750 mL containers at 3500 rpm for 5 min (Jouan C4-12 centrifuge, St Herblain, France). The time between cell harvest and the addition of the cells to the SSF process was less than 2 h.

### Prehydrolysis and simultaneous saccharification and fermentation

All SSF experiments were run in 2 L stirred tank reactors at a WIS concentration of 13.7% WIS, except SSF 3 (Table 1), which was run on the liquid fraction of the steam-pretreated slurry (at a concentration corresponding to 13.7% WIS). SSF 1, 4 and 8-10 were run on the whole pretreated slurry, while SSF 2 and 5-7 were run on washed pretreated slurry. Before the material was added to the fermentor, water was added to adjust the WIS concentration, and the pH was adjusted to 5 with NaOH. The reactors were then autoclaved together with the material at 121°C for 20 min. Nutrients were added to the reactors to give the following final concentrations: 0.5 g/L (NH_4_)_2_HPO_4_, 0.025 g/L MgSO_4_∙7H_2_O, and 1.0 g/L yeast extract. The commercial cellulase mixture Cellic CTec2 (108.9 FPU/ml and 4465 β-glucosidase IU/ml), kindly supplied by Novozymes A/S (Bagsværd, Denmark), was added at an amount corresponding to a cellulase activity of 10 FPU/g WIS in SSF 1-5, 7 and 9. SSF 6, 8 and 10 were run without the addition of cellulolytic enzymes. The initial concentration of fermentable sugars was adjusted to that presented in Table 1 by the addition of glucose. The SSF experiments were started by adding yeast at a concentration of 5 g dry yeast cells/L. All SSF experiments were run for 120 hours at a temperature of 32°C and a stirring speed of 700 rpm. The pH was continuously adjusted to 5 with 2.5 M NaOH.

In SSF 4 and 9, prehydrolysis was run at 48°C for 4 hours (SSF 9) and 22 hours (SSF 4) prior to the addition of yeast, when the temperature was lowered to 32°C. In these experiments, the time of yeast addition is defined as time 0.

### Analysis

Dry matter contents were determined by drying the samples in an oven at 105°C until constant weight was obtained. Concentrations of WIS were determined using the no-wash method described by Weiss *et al.*[31]. The composition of the washed solids from the pretreated slurry was determined according to the National Renewable Energy Laboratory (NREL) procedure for determination of structural carbohydrates and lignin in biomass [32]. Solids were separated from the liquid fraction of the pretreated slurry by filtration, and washed with excess water before analysis. The hydrolysate from the pretreated slurry was analyzed regarding its content of sugars using the NREL procedure for determination of sugars, by-products, and degradation products in liquid fraction process samples [33], and furfural, HMF, glycerol and the organic acids acetic acid, lactic acid. The oligosaccharide concentration was determined as the difference in monomeric sugar concentration before and after acid hydrolysis.

All samples were filtered through a 0.2 μm filter before analysis to remove particles. Sugar contents were analyzed using a high-performance anion-exchange chromatograph with pulsed amperometric detection in an ICS-3000 chromatography system (Dionex, Sunnyvale, CA, USA). A CarboPac PA1 guard column and a PA1 analytical column (Dionex) were used. Water was used as eluent at a flow rate of 1 mL/min, and 200 mM NaOH was added at a flow rate of 0.5 mL/min before the detector. The column was cleaned with 200 mM NaOH dissolved in 170 mM sodium acetate. The injection volume was 10 μL.

The samples taken from the SSF experiments and the liquid fraction of the pretreated slurry were also analyzed regarding their contents of lactic acid, acetic acid, HMF, furfural, glycerol and ethanol using HPLC with a refractive index detector (Shimadzu, Kyoto, Japan) and an Aminex HPX-87H column (Bio-Rad Laboratories, Hercules, CA), at 65°C, with 5 mM H_2_SO_4_ as eluent at a flow rate of 0.5 mL/min.

### Yield calculations

All ethanol yields were calculated using the measured amounts of total sugars in the solid and liquid fractions of the pretreated material and the fermentation broth at the end of SSF.

$$Y_{\mathrm{EtOH}}=\frac{c_{\mathrm{EtOH}}\left(1-\mathrm{WIS}\right)\frac{M}{1000}}{0.51\cdot \left[\mathrm{WIS}\cdot M\left(\sigma_{\mathrm{glc}}+\sigma_{\mathrm{man}}\right)+V_{\mathrm{hyd}}\left(c_{\mathrm{glc}}+c_{\mathrm{man}}\right)+m_{\mathrm{glc}+\mathrm{man}}\right]}$$

where Y_EtOH_ is the overall ethanol yield resulting from prehydrolysis and SSF (%), c_EtOH_ is the final concentration of ethanol (g/L,) WIS is the fraction of water-insoluble solids (%), M is the total mass (g), σ_glc_ and σ_man_ are the mass fractions of glucose and mannose in the pretreated fibers (g/g), V_hyd_ is the starting volume of hydrolysate in the reactor (L), c_glc_ and c_man_ are the concentrations of glucose and mannose in the hydrolysate (g/L), and m_glc+man_ is the amount of glucose and mannose added to the fermentor (g). The density of the liquid fraction was approximated to 1000 g/L. The exact fraction of liquid at the end of fermentation was not measured, and the starting volume was used for yield calculations. This results in a conservative yield calculation, with the ethanol yields presented being the lowest possible.

## Competing interests

The authors declare that they have no competing interests.

## Authors’ contributions

KH planned and carried out the experiments, analyzed the results and wrote the paper. GZ and MG participated in the design of the study, discussion of the results and finalization of the manuscript. All authors read and approved the final manuscript.
